# Effects of general anesthesia on short-term outcomes of patients with acute ischemic stroke after endovascular treatments: a meta-analysis

**DOI:** 10.3389/fneur.2025.1728140

**Published:** 2026-01-12

**Authors:** Shengshou Ye, Meiling Mao, Yun Zhang

**Affiliations:** Department of Neurology, Qinghai Cardiocerebrovascular Disease Specialized Hospital, Xining, China

**Keywords:** acute ischemic stroke, endovascular treatment, functional independence, general anesthesia, successful angiographic reperfusion

## Abstract

The optimal anesthetic strategy for endovascular treatment (EVT) in acute ischemic stroke (AIS) remains uncertain. This meta-analysis of randomized controlled trials (RCTs) compared the effects of general anesthesia (GA) and non-general anesthesia (non-GA) on reperfusion success and clinical outcomes after EVT. A systematic search of PubMed, Embase, Web of Science, and the Cochrane Library was performed. Eligible RCTs compared GA versus non-GA in adults with AIS undergoing EVT. The primary outcome was successful angiographic reperfusion (modified Thrombolysis in Cerebral Infarction 2b–3). Secondary outcomes included functional independence (modified Rankin Scale 0–2) and all-cause mortality at 3 months. Risk ratios (RRs) with 95% confidence intervals (CIs) were pooled using a random-effects model accounting for the influence of potential heterogeneity. Eleven RCTs encompassing 1,674 patients were included. Compared with non-GA, GA significantly increased the likelihood of successful reperfusion (RR = 1.08, 95% CI 1.02–1.13; I^2^ = 35%). However, GA did not significantly affect functional independence (RR = 1.11, 95% CI 0.98–1.26; I^2^ = 15%) or all-cause mortality (RR = 1.00, 95% CI 0.81–1.24; I^2^ = 0%). Subgroup analyses showed consistent results across vascular territories and baseline NIHSS strata. The certainty of evidence was moderate for reperfusion and low for functional and mortality outcomes. In conclusion, although GA improved angiographic reperfusion, this did not translate into improved 90-day functional outcomes. These findings support individualized anesthetic selection based on procedural and patient factors rather than routine GA use.

**Systematic review registration:**

https://www.crd.york.ac.uk/prospero/search, identifier CRD420251169607.

## Introduction

Acute ischemic stroke (AIS) remains a major cause of morbidity and mortality worldwide, with increasing incidence in aging populations (1, 2). Endovascular treatment (EVT) has become the standard of care for AIS caused by large-vessel occlusion, offering rapid mechanical recanalization and improved functional outcomes compared with medical therapy alone (3, 4). However, the optimal anesthesia modality during EVT—general anesthesia (GA) versus non-general anesthesia (non-GA, including conscious sedation or local anesthesia)—remains a topic of ongoing debate (5). GA can ensure airway protection and minimize patient motion but may cause hemodynamic instability and treatment delay, while non-GA allows for neurological monitoring but may risk agitation or conversion to GA (6, 7). These physiological and procedural differences have raised questions about their influence on both reperfusion success and short-term functional outcomes.

Several randomized controlled trials (RCTs) have directly compared GA and non-GA during EVT, but their findings have been inconsistent (8, 9). Some studies reported improved reperfusion (10–13) and functional independence (14) with GA, while others found no difference (15–18) or even a trend toward better outcomes with non-GA (19). Previous meta-analyses synthesizing these trials also yielded conflicting results, particularly regarding functional dependency and mortality, likely due to variations in inclusion criteria, small sample sizes, crossover rates, and heterogeneous sedation protocols (8, 9, 20–24). Moreover, several recently published RCTs after 2023, including multicenter and posterior-circulation studies, have not been consistently incorporated into earlier reviews (13, 18, 19, 25). Therefore, we conducted an updated meta-analysis of RCTs to provide the most comprehensive and up-to-date evidence on the comparative effects of GA versus non-GA during EVT for AIS, focusing on angiographic reperfusion, functional independence, and mortality.

## Methods

This meta-analysis was conducted in accordance with the methodological standards outlined in the PRISMA (Preferred Reporting Items for Systematic Reviews and Meta-Analyses) statement (26) and the Cochrane Handbook for Systematic Reviews of Interventions (27). The study protocol was prospectively registered in the PROSPERO database (registration ID: CRD420251169607).

### Study inclusion and exclusion criteria

This meta-analysis included studies that met the inclusion criteria specified in the PICOS principle.

Population (P): Adult patients (≥18 years) with AIS undergoing EVT.

Intervention (I): GA administered during EVT. GA was defined as anesthesia involving airway control (endotracheal intubation or laryngeal mask) with administration of anesthetic agents resulting in loss of consciousness. For study eligibility, an “adequate” description of the intervention required explicit identification of GA versus non-GA as the randomized strategy. Because our primary objective was to compare anesthesia modality rather than specific anesthetic regimens, studies were not excluded for incomplete reporting of maintenance drugs (e.g., propofol vs. volatile agents) or intraoperative physiological targets (e.g., ETCO₂, blood pressure). This approach ensured that potentially relevant RCTs were not missed, although we recognize that protocol-level differences may influence outcomes.

Comparator (C): Non-GA approaches, including conscious sedation (CS) or monitored anesthesia care (MAC) etc.

Outcomes (O): Successful angiographic reperfusion, defined as modified Thrombolysis in Cerebral Infarction (mTICI) grade 2b–3 after EVT; Functional independence at 3 months, defined as a modified Rankin Scale (mRS) score of 0–2; All-cause mortality at 3 months.

Study design (S): Randomized controlled trials (RCTs) directly comparing GA and non-GA during EVT for AIS.

Studies were excluded if they: (1) were non-randomized, crossover, or observational; (2) did not clearly distinguish GA from non-GA, or if crossover between groups was so extensive that the randomized anesthesia strategies could no longer be interpreted as distinct (e.g., bidirectional crossover or conversion rates approaching the size of one arm); (3) lacked sufficient data for outcome extraction; (4) enrolled pediatric or animal subjects; or (5) were conference abstracts, reviews, editorials, or duplicate publications of the same trial. For studies with overlapping patients, the one with the largest sample size was included for the meta-analysis.

### Database search

PubMed, Embase, Web of Science, and Cochrane Library databases were searched using the combination of the following terms: (1) “general anesthesia” OR “conscious sedation” OR “monitored anesthesia care”; (2) “ischemic stroke” OR “stroke” OR “cerebral infarction” OR “brain infarction” OR “cerebrovascular infarction”; (3) “endovascular therapy” OR “mechanical thrombectomy” OR “thrombectomy” OR “intra-arterial thrombolysis” OR “endovascular thrombectomy” OR “neurointervention” OR “endovascular treatment” OR “contact aspiration” OR “endovascular” OR “stent” OR “intra-arterial” OR “intraarterial”; and (4) “random” OR “randomized” OR “randomly” OR “allocated” OR “control” OR” randomised” OR “allocation.” We included only full-text articles involving human participants that were published in peer-reviewed journals in the English language. To ensure comprehensive coverage, the reference lists of relevant reviews and original studies were also examined. The most recent literature search was completed on October 14, 2025. The full search strategy for each database is shown in Supplemental file 1.

### Data extraction and quality evaluation

Two reviewers independently performed the literature search, data extraction, and quality assessment. Any discrepancies were resolved through discussion with the corresponding author. Extracted data included general study information (first author, publication year, and country), study design (double-blind, single-blind, or open-label), patient characteristics (diagnosis, sample size, mean age, sex, and baseline National Institutes of Health Stroke Scale [NIHSS]), details of the intervention (GA), comparator (non-GA), endovascular procedure, and reported outcomes. The methodological quality of included randomized controlled trials was evaluated using the Cochrane Risk of Bias 2.0 (RoB 2) tool, which assesses five domains: randomization process, deviations from intended interventions, missing outcome data, measurement of outcomes, and selection of reported results (27). Each domain was rated as low risk, some concerns, or high risk of bias, leading to an overall judgment. Additionally, two authors independently appraised the certainty of evidence using the GRADE (Grading of Recommendations, Assessment, Development and Evaluation) approach, which considers risk of bias, inconsistency, indirectness, imprecision, and publication bias (28). Evidence certainty was classified as very low, low, moderate, or high, with disagreements resolved through consensus.

### Statistical analysis

The effects of GA versus non-GA on successful angiographic reperfusion, functional independence, and all-cause mortality at 3 months were expressed as risk ratios (RRs) with corresponding 95% confidence intervals (CIs) (27). Between-study heterogeneity was evaluated using Cochran’s Q test and quantified with the I^2^ statistic, where values < 25%, 25–75, and >75% indicated low, moderate, and high heterogeneity, respectively (29). A random-effects model was applied to account for potential variability across trials (27). Leave-one-out sensitivity analysis was performed to test the robustness of pooled results (27). Prespecified subgroup analyses were conducted according to the vascular territory of stroke (anterior vs. posterior circulation) and the mean baseline NIHSS, using median values as cutoffs to ensure balanced subgroup sizes. Publication bias was assessed visually using funnel plots and statistically with Egger’s regression test (30). A two-tailed *p* value <0.05 was considered significant. All analyses were conducted using RevMan (version 5.3, Cochrane Collaboration, Oxford, United Kingdom) and Stata (version 17.0, StataCorp, College Station, TX, United States).

## Results

### Literature search

**Figure 1:**
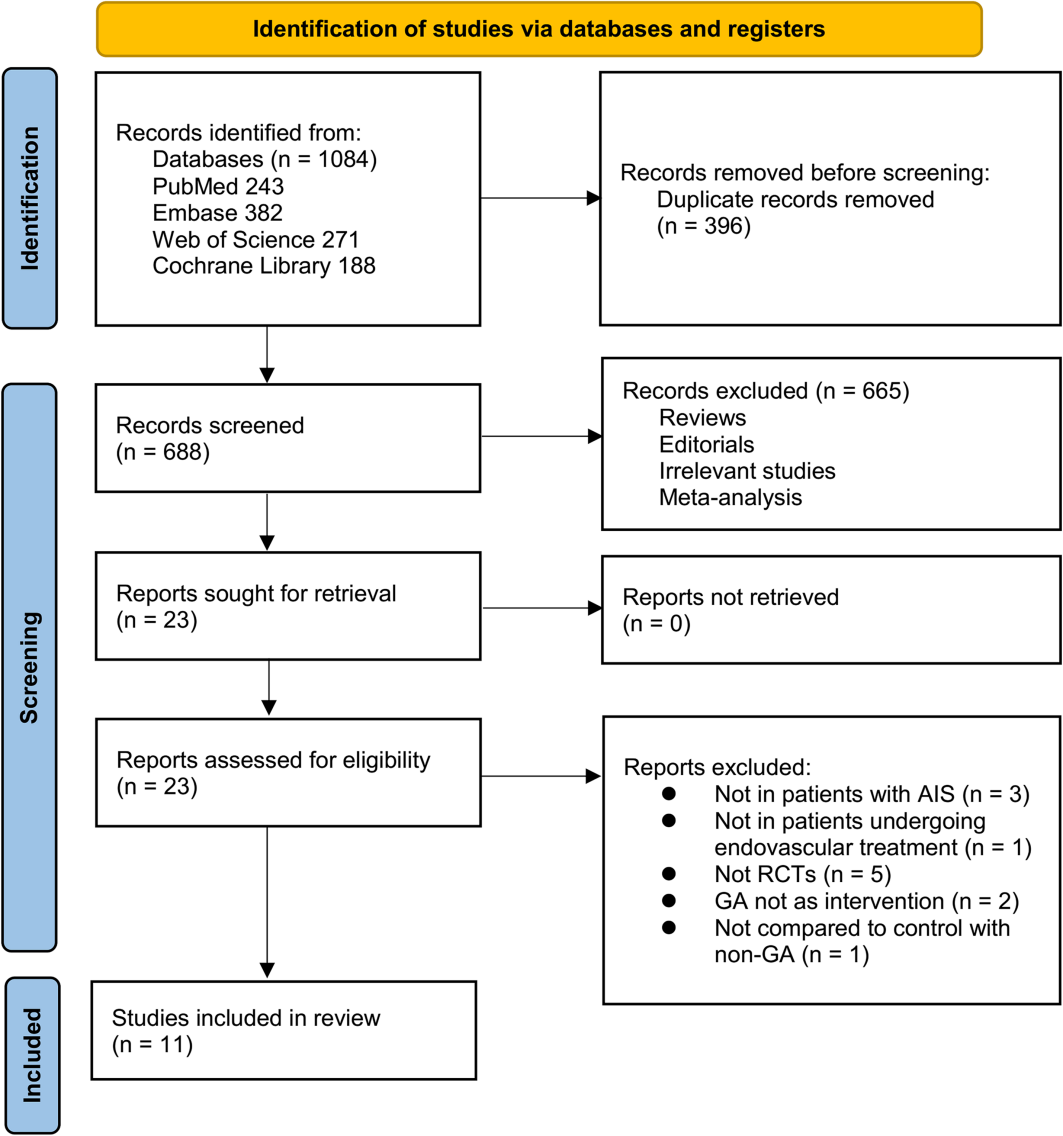
Flowchart for the literature search and study inclusion.

Figure 1 depicts the flowchart that outlines the process of database searching and study identification, ultimately leading to the selection of studies for inclusion. Initially, a total of 1,084 articles were obtained through the database search, which was subsequently reduced to 688 after eliminating duplicate records. Subsequently, 665 articles were excluded based on an evaluation of their titles and abstracts, primarily due to their lack of relevance to the objective of the present meta-analysis. The remaining 23 full-text articles were assessed, and 12 were excluded for reasons detailed in Figure 1. Ultimately, 11 RCTs (10–19, 25) were deemed suitable for quantitative analysis.

### Study characteristics

An overview of the included RCTs is presented in Table 1. This meta-analysis incorporated 11 RCTs published between 2016 and 2025, conducted across Europe (Germany, Sweden, Denmark, and France), the United States, and Asia (China). Collectively, these studies enrolled 1,674 patients with acute ischemic stroke (AIS) caused by large vessel occlusion (LVO) in either the anterior (internal carotid or middle cerebral arteries) (10–12, 14–16, 18, 19, 25) or posterior circulation (vertebrobasilar system) (13, 17). The mean age of participants ranged from 62.0 to 72.5 years, and the proportion of male patients varied between 48.0 and 81.6%. Baseline NIHSS scores were typically moderate to severe, ranging from 13.5 to 18.5. All studies compared GA with either CS (10–16, 18, 19, 25) or MAC (17) administered during EV). GA protocols commonly involved endotracheal intubation and mechanical ventilation, with induction using propofol, remifentanil, and neuromuscular blockers, followed by maintenance with propofol and/or volatile anesthetics. In contrast, non-GA approaches employed intravenous sedatives (propofol, remifentanil, or dexmedetomidine) without airway instrumentation, maintaining spontaneous respiration. EVT was performed using stent retriever and/or direct aspiration techniques, with procedural details generally left to operator discretion. All included trials reported at least one of the three predefined outcomes—successful angiographic reperfusion (mTICI 2b–3) in all the 11 studies (10–19, 25), functional independence at 3 months (mRS 0–2) in 10 studies (10–15, 17–19, 25), and all-cause mortality at 3 months in 10 studies (10–16, 18, 19, 25).

**Table 1:** Characteristics of the included studies.

Study	Country	Design	Diagnosis	No. of patients	Mean age (years)	Men (%)	Baseline mean NIHSS	Details of GA	Details of control	Details of EVT	Outcomes reported
Schönenberger 2016	Germany	R, OL	AIS in the anterior circulation (ICA or MCA occlusion), NIHSS > 10	150	71.5	60	17	GA with endotracheal intubation. Used intravenous, low-dose, short-acting analgesics and sedatives, at higher doses or with alternative/additional medications compared to the CS group	CS. Not intubated. Received intravenous, low-dose, short-acting analgesics and sedatives	Thrombectomy technique (stent retriever or direct aspiration) at interventionist’s discretion	Successful recanalization, functional independence at 3 months, and all-cause mortality at 3 months
Hendén 2017	Sweden	R, OL	AIS in the anterior circulation (ICA or MCA occlusion). NIHSS ≥10 (right-sided) or ≥14 (left-sided)	90	72.5	54.4	18.5	GA with endotracheal intubation. Induced with propofol and remifentanil, maintained with sevoflurane and remifentanil. Normoventilation was targeted	CS. Not intubated. Sedation performed by remifentanil infusion	Embolectomy techniques (direct aspiration, stentrievers, or snare) at interventionist’s discretion	Successful recanalization, functional independence at 3 months, and all-cause mortality at 3 months
Simonsen 2018	Denmark	R, OL	AIS in the anterior circulation (Large Vessel Occlusion). Within 6 h of onset. Baseline infarct volume <70 mL on MRI-DWI	128	71.4	51.6	17.5	GA with endotracheal intubation. Protocol aimed to limit time delay and maintain BP (SBP > 140 mm Hg, MAP >70 mm Hg). Propofol was used	CS. Not intubated. Sedation also performed using propofol to minimize drug confounding	Thrombectomy technique (stent retriever, direct aspiration, or combination) at interventionist’s discretion	Successful recanalization, functional independence at 3 months, and all-cause mortality at 3 months
Ren 2020	China	R, OL	AIS in the anterior circulation (ICA, M1/M2, A1). Within 6.5 h of onset. NIHSS < 20	90	70.4	56.7	14.6	GA with endotracheal intubation. Induced with propofol, fentanyl, cisatracurium. Maintained with propofol, remifentanil, dexmedetomidine, cisatracurium	CS. Not intubated. Used propofol and dexmedetomidine, with supplemental fentanyl or midazolam.	Mechanical thrombectomy (stent retriever or direct aspiration) at interventionist’s discretion	Successful recanalization, and all-cause mortality at 3 months
Sun 2020	China	R, OL	AIS due to anterior circulation large vessel occlusion (ICA, M1/M2 MCA)	40	63.5	65	13.5	GA with laryngeal mask or endotracheal tube + mechanical ventilation. Induction: Sufentanil 0.2 μg/kg, Propofol TCI 1–4 μg/mL, Rocuronium 0.6 mg/kg. Maintenance: Propofol TCI 1–4 μg/mL, Remifentanil 0.1–0.2 μg/kg/min. BIS target 40–60	CS. Supplemental O₂ via facemask. Sufentanil 0.1 μg/kg bolus + Propofol TCI 0.5–1.0 μg/mL. BIS target >70	Mechanical thrombectomy using direct aspiration, stent retriever, or a combination	Successful recanalization, functional independence at 3 months, and all-cause mortality at 3 months
Hu 2021	China	R, OL	AIS due to vertebrobasilar occlusion (posterior circulation)	139	72	51.8	NR	GA with endotracheal intubation + mechanical ventilation. Induction: Suxamethonium 0.5–1 mg/kg, Alfentanil 0.02–0.03 mg/kg, Propofol 1–5 mg/kg. Maintenance: Propofol 2–10 mg/kg/h, Remifentanil 0.2–1 μg/kg/min.	MAC: Fentanyl bolus 25–50 μg (repeated as needed) + Propofol infusion 1–4 mg/kg/h. Target: Ramsay sedation score of 4–5.	Mechanical thrombectomy using stent retriever, ADAPT technique (direct aspiration), or a combination	Successful recanalization, and functional independence at 3 months
Maurice 2022	France	R, SB	AIS due to large vessel occlusion in the anterior cerebral circulation (ICA, M1/M2 MCA)	343	71.7	55.4	16	GA with endotracheal intubation + mechanical ventilation. Induction: Etomidate (0.25–0.4 mg/kg), Succinylcholine (1 mg/kg). Maintenance: TCI Propofol (max target 4 μg/ml) and TCI Remifentanil (0.5–4 ng/mL).	CS. TCI Remifentanil (max target 2 ng/mL) + Local anesthesia with lidocaine. Oxygen administered only if SpO₂ ≤ 96%.	Mechanical thrombectomy	Successful recanalization, functional independence at 3 months, and all-cause mortality at 3 months
Liang 2023	China	R, OL	AIS due to large vessel occlusion in the posterior circulation (basilar artery or vertebral artery)	87	62	81.6	15	GA with endotracheal intubation + mechanical ventilation. Induction: Propofol (1–2 mg/kg), Remifentanil (0.2–0.8 μg/kg/min), Rocuronium (0.6 mg/kg).	CS. Propofol bolus (0.3–0.5 mg/kg) then continuous infusion of Propofol (1–2 mg/kg/h) and Remifentanil (0.01–0.06 μg/kg/min).	Endovascular thrombectomy	Successful recanalization, functional independence at 3 months, and all-cause mortality at 3 months
Chabanne 2023	France	R, OL	AIS due to anterior circulation large-vessel occlusion (intracranial ICA and/or proximal M1/M2 MCA)	273	71.6	48	16	GA with endotracheal intubation. Mechanical ventilation with EtCO₂ maintained at 30–35 mm Hg. Choice of anesthetic agents followed local protocols.	CS. Goal RASS 0 to −3.	Mechanical thrombectomy (stent retriever and/or thrombus aspiration) following local protocols and established guidelines	Successful recanalization, functional independence at 3 months, and all-cause mortality at 3 months
Zhang 2024	China	R, OL	AIS due to anterior circulation large vessel occlusion. Onset <6 h. NIHSS ≥6.	90	64.1	58.9	15.9	GA with endotracheal intubation. Induction: Remifentanil (0.5–1 μg/kg), Propofol (TCI, target 2 μg/ml), Cis-atracurium (0.1–0.2 mg/kg). Maintenance: Remifentanil (0.05–0.1 μg/kg/min) + Propofol (TCI, target 2–3 μg/mL). Laryngeal mask, mechanical ventilation. BIS target 40–60.	CS. Propofol (TCI, target 0.5–1.0 μg/ml) + Sufentanil (5–10 μg) or Remifentanil (0.03–0.05 μg/kg/min). BIS maintained >70.	Stent retriever thrombectomy, direct aspiration, or arterial thrombolysis.	Successful recanalization, functional independence at 3 months, and all-cause mortality at 3 months
Chen 2025	USA	R, OL	AIS due to anterior circulation large-vessel occlusion (ICA terminus, MCA M1/proximal M2, ACA A1/proximal A2)	244	66.8	52	15	GA with endotracheal intubation. Induction: Propofol and/or etomidate, with succinylcholine or a nondepolarizing paralytic. Maintenance: Either intravenous (Propofol infusion) or inhalational (Sevoflurane or Desflurane).	CS. Target RASS -1 to −3. Medications: Fentanyl, Midazolam, Dexmedetomidine, and/or low-dose Propofol.	Mechanical thrombectomy following AHA/ASA guidelines, using stent-retriever devices or direct aspiration catheters.	Successful recanalization, functional independence at 3 months, and all-cause mortality at 3 months

AIS, acute ischemic stroke; ACA, anterior cerebral artery; ADAPT, A Direct Aspiration First Pass Technique; AHA, American Heart Association; ASA, American Stroke Association; BIS, bispectral index; BP, blood pressure; CS, conscious sedation; DWI, diffusion-weighted imaging; EtCO₂, end-tidal carbon dioxide; EVT, endovascular treatment; GA, general anesthesia; ICA, internal carotid artery; LMA, laryngeal mask airway; LVO, large vessel occlusion; MAC, monitored anesthesia care; MAP, mean arterial pressure; MCA, middle cerebral artery; mRS, modified Rankin Scale; mTICI, modified Thrombolysis in Cerebral Infarction; MRI, magnetic resonance imaging; NIHSS, National Institutes of Health Stroke Scale; NR, not reported; OL, open-label; R, randomized; RASS, Richmond Agitation-Sedation Scale; SB, single-blind; SBP, systolic blood pressure; SpO₂, peripheral capillary oxygen saturation; TCI, target-controlled infusion.

### Study quality assessment

As summarized in Table 2, the methodological quality of the included RCTs was generally acceptable, with all studies rated as “some concerns” overall according to the Cochrane Risk of Bias 2.0 (RoB 2) tool. Randomization procedures were adequately described and implemented in most studies (e.g., web-based or computer-generated allocation with concealment), though several trials provided limited details (15, 17, 18). All studies were open-label, as anesthesia type could not be blinded to treating clinicians, introducing potential performance bias. However, the primary outcomes were consistently assessed by blinded evaluators, mitigating detection bias. Crossovers from CS to GA occurred in all trials but were generally below 20% and managed using intention-to-treat analyses. Outcome data were nearly complete across all trials, with follow-up rates exceeding 90%, and selective reporting was unlikely, as most trials had pre-registered protocols. Overall, the evidence base comprises well-conducted multicenter RCTs with rigorous blinding of outcome assessment and minimal attrition, supporting the reliability of the pooled results while acknowledging the inherent limitations of open-label anesthesia comparisons.

**Table 2:** Risk bias evaluation via Cochrane risk of bias tool 2.0 with reasons.

RCTs	Randomization process	Deviations from intended interventions	Missing outcome data	Measurement of the outcome	Selection of the reported results	Overall
Schönenberger 2016	Low risk: Participants were randomized 1:1 using a computer-generated list and sealed opaque envelopes. Baseline characteristics were well-balanced between groups.	Some concerns: The trial was open-label (participants and care providers were aware of the assignment). While the analysis was by intention-to-treat, the knowledge of assignment could have influenced peri-procedural management (e.g., hemodynamic control). Furthermore, there was one major protocol violation (1 patient in GA group received CS) and a 14.3% crossover from CS to GA. Although this reflects clinical practice, it introduces concerns regarding the effect of assignment to intervention.	Low risk: Follow-up for the primary outcome (24-h NIHSS) was nearly complete (100% in GA, 99% in CS). The 3-month functional outcome assessment also had very low attrition, unlikely to bias the results.	Low risk: The assessors of the primary outcome (NIHSS at 24 h) and the key secondary outcome (mRS at 3 months) were blinded to the treatment allocation.	Low risk: The study protocol was published previously, and all pre-specified outcomes (primary and 47 secondary outcomes) appear to have been reported.	Some concerns
Hendén 2017	Some concerns: Participants were randomized using sealed, opaque envelopes. However, there is a notable baseline imbalance in the NIHSS score, which was higher in the GA group (median 20 vs. 17), and the proportion of left-sided occlusions (58% vs. 38%). This suggests that the randomization process may not have been fully effective in creating balanced groups, potentially due to the small sample size.	Some concerns: The trial was open-label. There was a 15.6% crossover (7/45 patients) from the CS group to the GA group. Although an intention-to-treat analysis was used, the knowledge of the assignment and the crossovers introduce some concerns.	Low risk: No patients were lost to follow-up for the primary outcome (3-month mRS). All 90 randomized patients were analyzed.	Low risk: The assessment of the primary outcome (mRS at 3 months) was performed by a vascular neurologist who was blinded to the treatment allocation.	Low risk: The trial was registered (NCT01872884), and all pre-specified outcomes, including the primary outcome (mRS at 3 months), appear to have been reported.	Some concerns
Simonsen 2018	Low risk: Participants were randomized 1:1 using a web-based program with stratification by age and NIHSS score. Block randomization was used. Baseline characteristics were well-balanced between groups.	Some concerns: The trial was open-label. There was a 6.3% crossover (4/63 patients) from the CS group to the GA group. Although an intention-to-treat analysis was used, the knowledge of the assignment and the crossovers introduce some concerns.	Low risk: Only one patient refused post-randomization consent and was excluded. Follow-up for the 90-day mRS (the key outcome for this meta-analysis) was complete for the remaining 127 patients (99.2%).	Low risk: The assessment of the 90-day mRS score was performed by a certified study nurse who was blinded to the randomization group.	Low risk: The trial was registered (NCT02317237), and the protocol was published previously. All pre-specified outcomes appear to have been reported.	Some concerns
Ren 2020	Low risk: A computer-generated randomization table was used by an independent assistant. Baseline characteristics were comparable between groups.	Some concerns: The trial was open-label. There was a 9.52% crossover (4/42 patients) from the CS group to the GA group. Although an intention-to-treat analysis was used, the knowledge of the assignment and the crossovers introduce some concerns.	Low risk: All 90 randomized patients were included in the analysis of the primary outcome (90-day mRS). No patients were lost to follow-up.	Low risk: The assessors of the primary outcome (90-day mRS) and secondary outcomes (NIHSS, etc.) were blinded to the group allocation.	Some concerns: The trial was registered (ChiCTR-IPR-16008494). The primary outcome (mRS 0–2 at 90 days) is clearly reported. However, the results for the primary outcome are only presented as “not significantly different” without providing the raw numbers.	Some concerns
Sun 2020	Low risk: Randomization was performed through a purposely built web-based program, stratified by the site of culprit vessels using permuted blocks. This describes a robust randomization process.	Some concerns: This was an open-label trial. While the primary outcomes for the main trial are blinded (mRS assessed by certified neurologists blinded to allocation), the intraoperative management could be influenced by knowledge of the assigned intervention. Furthermore, there was a 18.2% (4/22) crossover from the CS group to the GA group. The authors performed both ITT and per-protocol analyses, which showed similar results for the main outcomes, somewhat mitigating this concern. However, the potential for bias from these deviations remains.	Low risk: The modified intention-to-treat analysis included 40 out of 43 randomized patients. The flow diagram (Figure 1) indicates the reasons for exclusion post-randomization, and the loss (3 patients) is unlikely to impact the outcome significantly.	Low risk: The critical outcome (90-day mRS) was assessed by “certified neurologists who were blinded to the group allocation.” This ensures a low risk of bias in outcome measurement.	Low risk: The trial was registered on ClinicalTrials.gov (NCT02677415), and a detailed protocol was published beforehand, suggesting that the reported results align with the pre-specified plan.	Some concerns
Hu 2021	Some concerns: The paper states patients “were assigned randomly” but provides no details on the method of random sequence generation or allocation concealment.	Some concerns: The trial was open-label. There was a crossover: 2 patients in the MAC group were converted to GA due to respiratory insufficiency and agitation. The authors state an intention-to-treat (ITT) analysis was used, which is good, but the potential for bias from these deviations in an open-label study remains.	Low risk: All 139 randomized patients appear to be included in the analysis, with no mention of missing outcome data for the primary 90-day mRS.	Low risk: The 90-day mRS was assessed “by a registered nurse who was unaware of the randomization by telephone.” This blinding of the outcome assessor ensures a low risk of bias for this domain.	Some concerns: There is no mention of a pre-published or registered protocol (e.g., on ClinicalTrials.gov).	Some concerns
Maurice 2022	Low risk: The paper describes a robust process: “centralized and computer generated,” with “block-randomization stratified by center, the NIHSS. and the administration (or not) of IV thrombolysis.” Treatment assignments were concealed from patients, outcome assessors, and the statistician.	Some concerns: This was an open-label trial for the clinicians. There was a pre-specified protocol for crossover (8 patients, 4.5%, crossed over from CS to GA). While an ITT analysis was used, the effect of knowing the intervention assignment on intraoperative management (e.g., hemodynamic control) could introduce bias. This is a common and often unavoidable concern in trials comparing different anesthesia techniques.	Low risk: Data on the primary outcome (90-day mRS) was available for 341/345 (98.8%) patients in the ITT population. This is a very low and unlikely-to-bias rate of missingness.	Low risk: The primary outcome (mRS at 3 months) was “assessed by trained research nurses blinded to the randomization group.” This ensures a low risk of bias.	Low risk: The trial was registered (NCT02822144), and a detailed protocol was published beforehand. The reported outcomes match those pre-specified in the methods section.	Some concerns
Liang 2023	Low risk: The paper describes a robust process: “a computer-generated random block number table was used (blocks of 4)” and allocation was implemented “through opaque, sealed, and stapled envelopes.”	Some concerns: This was an open-label trial for the clinicians. There was a very high and clinically important crossover rate: 13 of 44 patients (29.5%) in the CS group were converted to GA. The authors performed both ITT and per-protocol analyses, which is strength, but the high rate of deviation in an open-label trial introduces concerns about the effect of the intervention as assigned.	Low risk: Of the 93 randomized patients, 87 (93.5%) were included in the ITT analysis for the primary outcome. The reasons for exclusion are provided, and the rate is unlikely to introduce bias.	Low risk: The primary outcome (90-day mRS) was “evaluated by blinded researchers with a recorded telephone call.” This ensures a low risk of bias in outcome measurement.	Low risk: The trial was registered (NCT03317535), and a detailed protocol was published beforehand. The reported outcomes match those pre-specified.	Some concerns
Chabanne 2023	Low risk: The trial used a “password-protected web-based randomization system and minimization algorithm” with stratification, which is a robust method. Baseline characteristics were well-balanced.	Some concerns: This was an open-label trial. While the intervention (anesthesia type) cannot be blinded, the authors state that the protocol allowed for crossover from procedural sedation to GA (which occurred in 10.9% of patients). The effect of this deviation from the assigned intervention on the outcome is not fully known, leading to some concerns.	Low risk: The primary outcome was assessed in the modified intention-to-treat population (273/329, ~83%). For the 90-day mRS, a single blinded assessor conducted structured telephone interviews, and loss to follow-up was minimal (4 patients in the procedural sedation group, 0 in the GA group for the primary outcome assessment).	Low risk: The assessment of the primary outcome (functional independence) was performed by a “single certified trial investigator who was unaware of the group assignments,” ensuring blinded outcome assessment.	Low risk: The trial was registered (NCT03229148), and the statistical analysis plan was published prior, suggesting that the data analysis was consistent with a pre-specified plan.	Some concerns
Zhang 2024	Some concerns: The study does not detail the method used to generate the random sequence or the mechanism of allocation concealment.	Low risk: The analysis was performed on an Intention-to-Treat (ITT) basis, which is appropriate. The protocol clearly states that no patients crossed over from the CS group to the GA group. Performance bias is likely, as it was an open-label study, but the primary outcomes (mRS, mortality) are unlikely to be influenced by this knowledge.	Low risk: The text states “all cases were followed up,” and the flow diagram confirms that all 90 randomized patients were included in the analysis for the 90-day outcomes. There is no evidence of missing outcome data.	Low risk: The outcomes of successful recanalization (mTICI grade) and functional independence (mRS score) involve a degree of clinical judgment. While the assessors were likely not blinded to the treatment group (open-label design), the mRS is a well-established, standardized scale, and mTICI grading has defined criteria, making them less susceptible to measurement bias.	Low risk: The trial was registered (NCT02677415), and the statistical analysis plan was published prior, suggesting that the data analysis was consistent with a pre-specified plan.	Some concerns
Chen 2025	Low risk: The trial used a “secure web-based system using permuted blocks” with stratification, which is a robust method. Baseline characteristics were largely similar.	Some concerns: This was an open-label trial. There was a 7.7% crossover rate, primarily from sedation to GA (18/130, 13.8%) due to agitation. The effect of these deviations on the outcome is not fully known, leading to some concerns. A per-protocol analysis was conducted and showed similar results.	Low risk: For the primary outcome (90-day mRS), 240 of 260 randomized patients (92.3%) were included in the full-analysis population. Loss to follow-up was minimal (2 patients in the sedation group).	Low risk: The assessment of the primary outcome (90-day mRS) was performed by “certified study personnel blinded to anesthesia allocation.”	Low risk: The trial was registered (NCT03263117), and the statistical analysis plan used a pre-specified Bayesian framework. The reported outcomes match those listed in the methods section.	Some concerns

GA, general anesthesia; CS, conscious sedation; MAC, monitored anesthesia care; NR, not reported; NIHSS, National Institutes of Health Stroke Scale; mRS, modified Rankin Scale; mTICI, modified Thrombolysis in Cerebral Infarction; ITT, intention-to-treat; RCT, randomized controlled trial; IV, intravenous; OL, open-label; SB, single-blind.

### Meta-analysis results

**Figure 2:**
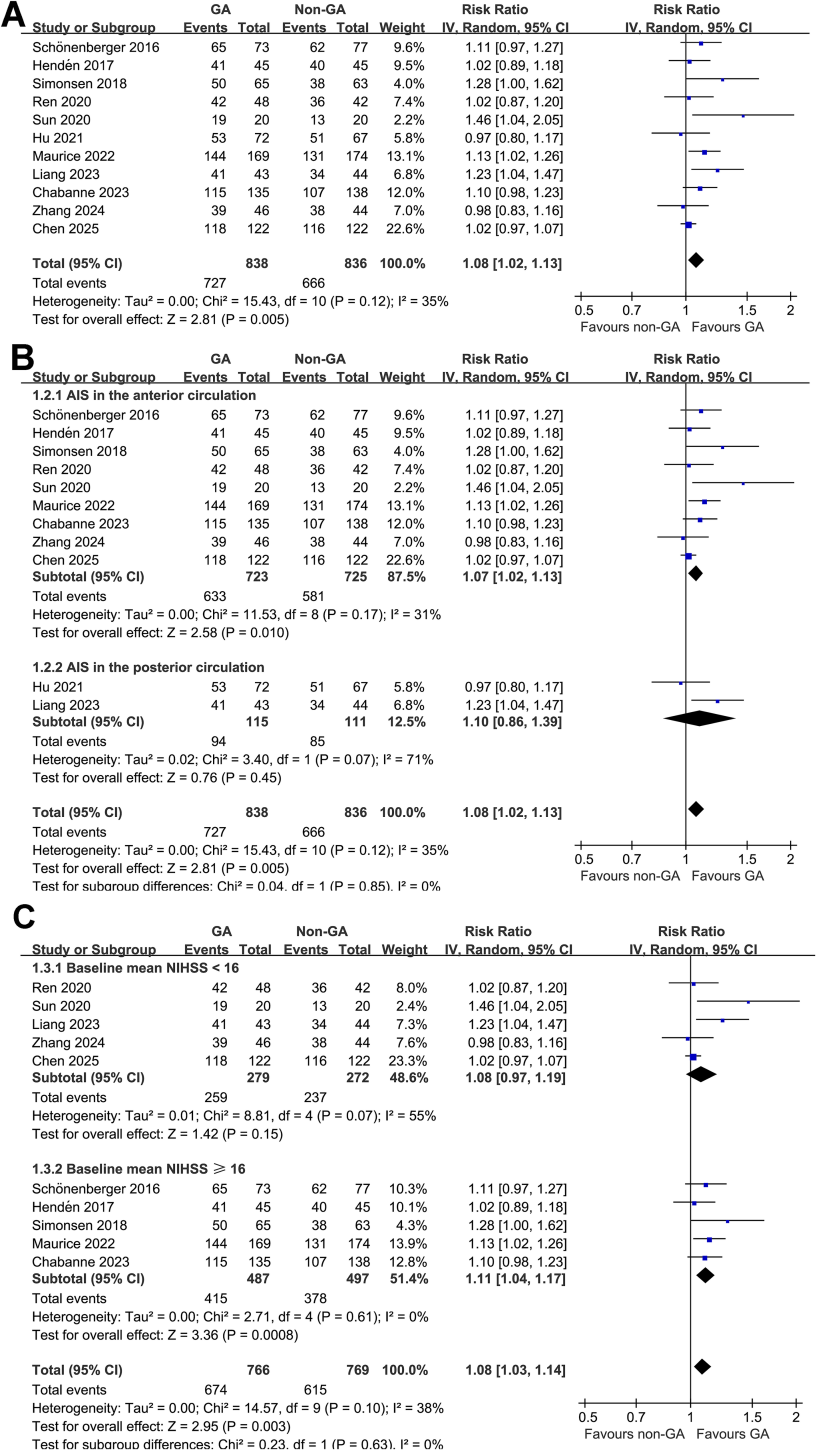
Forest plots for the meta-analysis evaluating the influence of GA on successful angiographic reperfusion (mTICI 2b-3) of AIS patients after EVT; **(A)** overall meta-analysis; **(B)** subgroup analysis according to the vascular territory of AIS; and **(C)** subgroup analysis according to the mean NIHSS at admission.

Pooled results of 11 RCTs (10–19, 25) showed that compared to non-GA, GA was associated with a significantly improved successful angiographic reperfusion after EVT in patients with AIS (RR: 1.08, 95% CI: 1.02 to 1.13, *p* = 0.005; Figure 2A) with moderate heterogeneity (*p* for Cochrane Q test = 0.12, I^2^ = 35%). Sensitivity analysis by excluding one study at a time showed consistent results (RR: 1.06 to 1.09, *p* all < 0.05). Moreover, the results were not significantly different between patients with AIS in the anterior and posterior circulation (RR: 1.07 vs. 1.10, *p* for subgroup difference = 0.85; Figure 2B), or between studies of patients with mean NIHSS at admission < 16 or ≥ 16 (RR: 1.08 vs. 1.11, *p* for subgroup difference = 0.63; Figure 2C).

**Figure 3:**
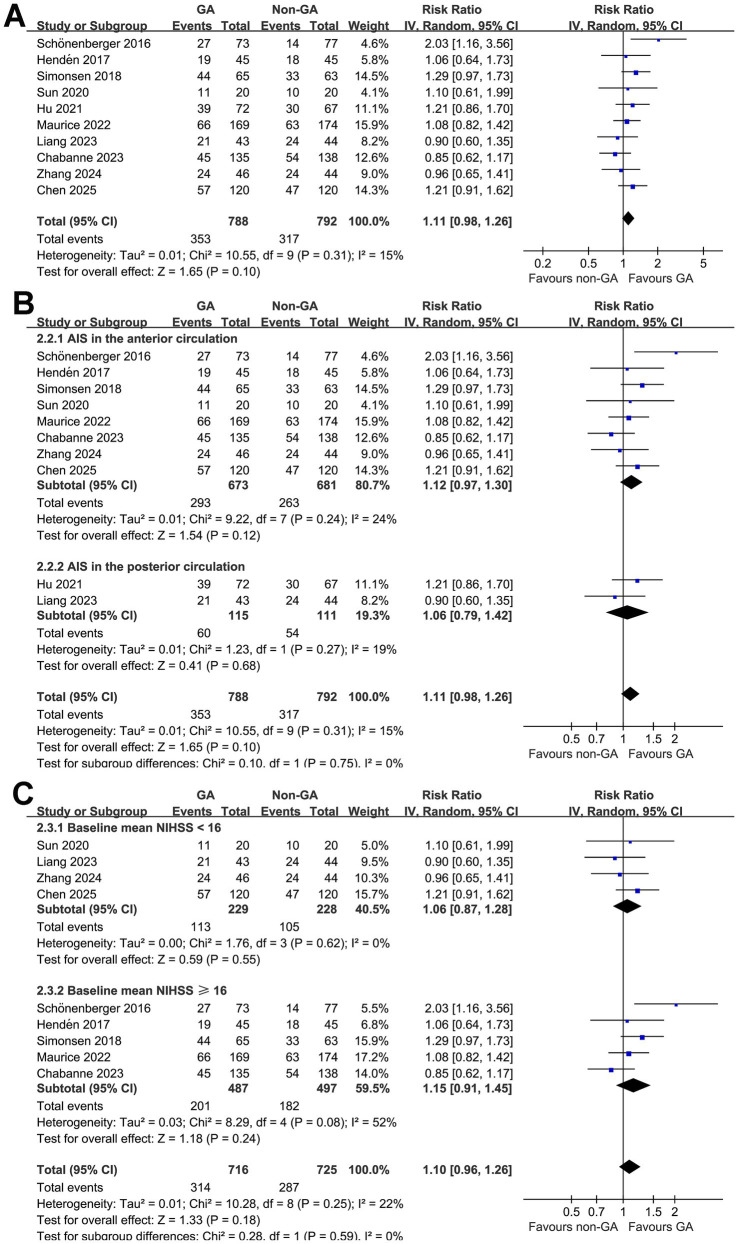
Forest plots for the meta-analysis evaluating the influence of GA on functional independence (mRS: 0–2) of AIS patients at 3 months after EVT; **(A)** overall meta-analysis; **(B)** subgroup analysis according to the vascular territory of AIS; and **(C)** subgroup analysis according to the mean NIHSS at admission.

Subsequent pooled analysis with 10 studies (10–15, 17–19, 25) did not show that GA significantly affect the outcome of functional independence at 3 months as compared to GA of AIS patients after EVT (RR: 1.11, 95% CI: 0.98 to 1.26, *p* = 0.10; Figure 3A) with mild heterogeneity (*p* for Cochrane Q test = 0.31, I^2^ = 15%). Sensitivity analysis by excluding one study at a time did not significantly change the results (RR: 1.08 to 1.15, *p* all > 0.05). Subgroup analysis according to the vascular territory of AIS and mean NIHSS at admission did not significantly change the results (*p* for subgroup difference = 0.76 and 0.58; Figures 3B,C).

**Figure 4:**
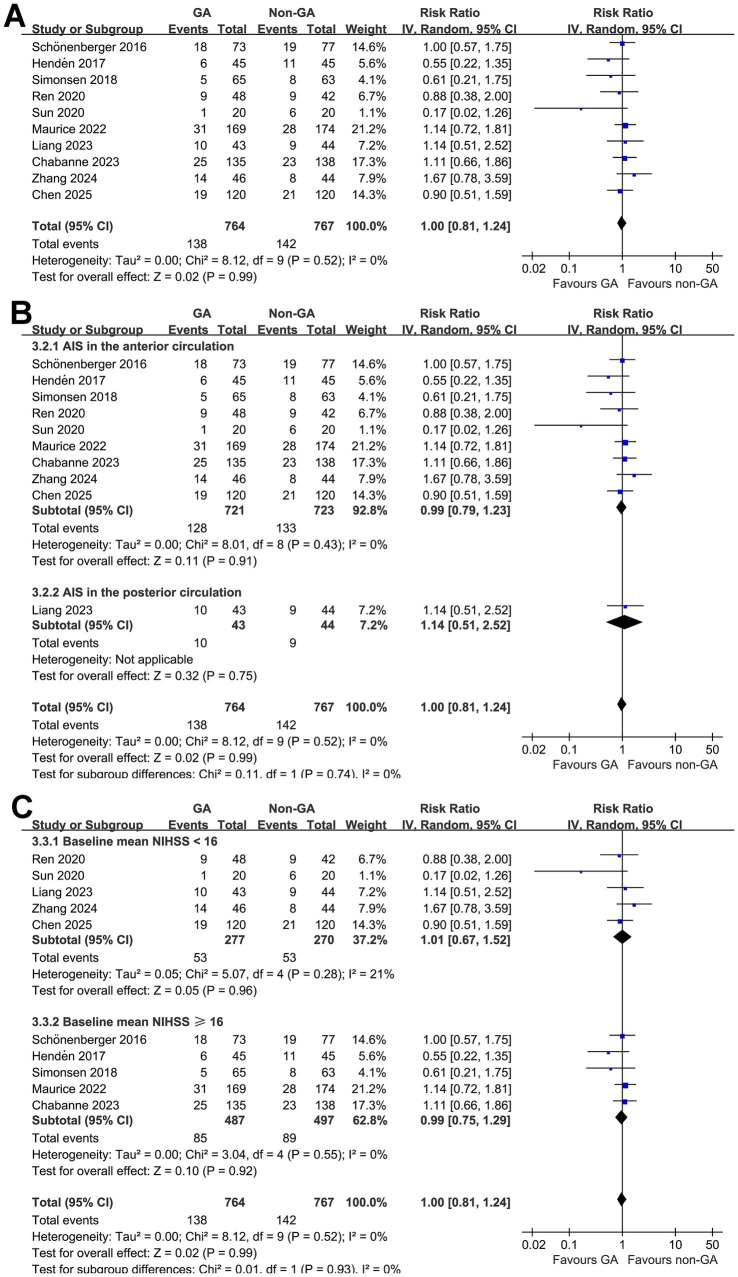
Forest plots for the meta-analysis evaluating the influence of GA on all-cause mortality of AIS patients at 3 months after EVT: **(A)** overall meta-analysis; **(B)** subgroup analysis according to the vascular territory of AIS; and **(C)** subgroup analysis according to the mean NIHSS at admission.

Finally, pooled results of 10 studies (10–16, 18, 19, 25) did not show a significant difference of all-cause mortality for AIS patients at 3 months after EVT (RR: 1.00, 95% CI: 0.81 to 1.24, *p* = 0.99; Figure 4A) with no significant heterogeneity (*p* for Cochrane Q test = 0.52, I^2^ = 0%). Sensitivity analysis by excluding one study at a time did not significantly change the results (RR: 0.96 to 1.0, *p* all > 0.05). Subgroup analysis according to the vascular territory of AIS and mean NIHSS at admission did not significantly affect the results (*p* for subgroup difference = 0.74 and 0.93; Figures 4B,C).

### Publication bias

**Figure 5:**
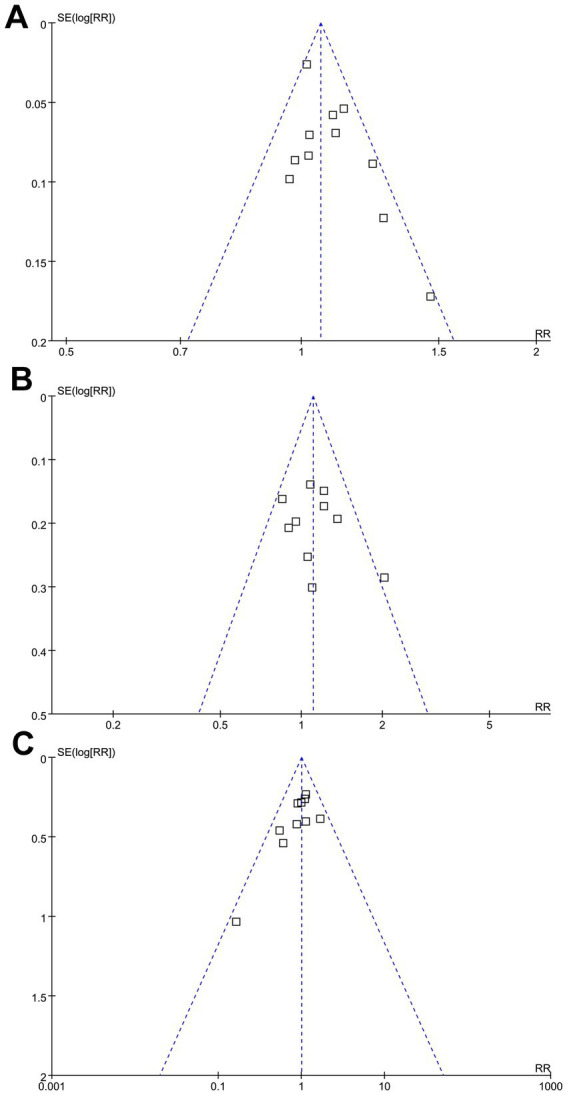
Funnel plots evaluating the publication bias underlying the meta-analyses: **(A)** funnel plots for the meta-analysis evaluating the influence of GA on successful angiographic reperfusion (mTICI 2b-3) of AIS patients after EVT; **(B)** funnel plots for the meta-analysis evaluating the influence of GA on functional independence (mRS: 0–2) of AIS patients at 3 months after EVT; and **(C)** funnel plots for the meta-analysis evaluating the influence of GA on all-cause mortality of AIS patients at 3 months after EVT.

The funnel plots for the meta-analyses evaluating the effects of GA on successful angiographic reperfusion, functional independence at 3 months, and all-cause mortality at 3 months of AIS patients after EVT are shown in Figures 5A–C. These plots are symmetrical on visual inspection, suggesting a low risk of publication bias. Egger’s regression test also indicated a low risk of publication bias (*p* = 0.34, 0.57, and 0.29, respectively).

### Certainty of evidence

A summary of the certainty of evidence evaluated using the GRADE approach is presented in Table 3. The certainty for the outcome of successful angiographic reperfusion was rated as moderate, downgraded by one level due to potential performance bias associated with open-label study designs. For functional independence and all-cause mortality at 3 months, the certainty of evidence was judged as low, reflecting downgrading for both risk of bias and imprecision. Although all included trials were randomized and used blinded outcome assessment, the open-label nature of anesthesia assignment and limited total sample size (below the optimal information size for firm conclusions) reduced overall certainty. No downgrading was applied for inconsistency, indirectness, or publication bias, as results were consistent and directly addressed the research question.

**Table 3:** Summary of findings and certainty of evidence (GRADE).

Outcome	No. of participants (studies)	Study design	Risk of bias	Inconsistency	Indirectness	Imprecision	Other considerations	Relative effect: RR (95% CI),	Certainty of evidence (GRADE)	Comments
Successful angiographic reperfusion (mTICI 2b–3)	1,674 (11 RCTs)	RCTs	Serious—open-label design but blinded outcome assessment; no crossover; unlikely to bias objective angiographic results	Not serious—direction of effect consistent across studies; low heterogeneity (I^2^ = 35%)	Not serious—interventions, populations, and outcomes directly aligned with the review question	Not serious—pooled CI excludes no effect; total sample >1,500 provides adequate information size	None	1.08 (1.02 to 1.13)	⨁⨁⨁◯ Moderate	GA increased the likelihood of successful reperfusion compared with non-GA.
Functional independence at 3 months (mRS 0–2)	1,580 (10 RCTs)	RCTs	Serious—open-label, blinded assessors; minimal attrition	Not serious—consistent direction of effect with modest heterogeneity (I^2^ = 15%)	Not serious—same population, intervention, and endpoint as review focus	Serious—95% CI crosses unity and total N (<2,000) below optimal information size for detecting a 10% relative effect; event count <300	None	1.11 (0.97 to 1.26)	⨁⨁◯◯ Low	No significant difference between GA and non-GA in functional recovery.
All-cause mortality at 3 months	1,531 (10 RCTs)	RCTs	Serious—open-label, blinded assessors; no crossover	Not serious—mortality direction consistent; heterogeneity low (I^2^ = 0%)	Not serious—mortality definition uniform (death ≤90 days) and directly relevant	Serious—wide CI including benefit and harm; total deaths <300 → below optimal information size	None	1.00 (0.81 to 1.24)	⨁⨁◯◯ Low	No evidence that anesthetic technique affects short-term mortality.

GA, general anesthesia; non-GA, non-general anesthesia; mTICI, modified Thrombolysis in Cerebral Infarction; mRS, modified Rankin Scale; RR, risk ratio; CI, confidence interval; RCT, randomized controlled trial. Specific reasons for each GRADE domain, including: Risk of bias: Downgraded if a significant proportion of studies had unclear or high risk of bias in key domains (e.g., random sequence generation, allocation concealment, or selective reporting). Inconsistency: Downgraded if substantial heterogeneity was observed (I^2^ > 50%) and could not be explained by subgroup analyses or meta-regression. Indirectness: Evaluated but not downgraded, as all included studies directly assessed the population and outcomes of interest. Imprecision: Downgraded if confidence intervals were wide, overlapping no effect, or if the overall sample size was small. Publication bias: Assessed using funnel plots and Egger’s test; downgraded if significant asymmetry suggested potential bias. Footnotes / Explanations: 1. Risk of bias (−1 level): All RCTs were open-label by necessity, but used proper randomization, intention-to-treat analysis, blinded outcome assessment, and no crossover; therefore, downgraded one level only. 2. Inconsistency (not serious): Forest plots (Figures 2–4) show consistent direction of effects with overlapping CIs and low I^2^ values; no unexplained heterogeneity. 3. Indirectness (not serious): Populations (AIS with LVO), interventions (GA vs non-GA), and outcomes (mTICI, mRS, mortality) directly address the review question; evidence judged directly applicable. 4. Imprecision (serious for mRS and mortality): For these outcomes, pooled 95% CIs crossed the line of no effect, and total N (~1,600) with <300 events fell below the optimal information size (OIS) threshold (~3,000 participants or ≥300 events) for a 10% relative effect, indicating uncertainty in the true estimate. 5. Other considerations: No evidence of publication bias; similar results between large and small studies.

## Discussion

The present meta-analysis provides a comprehensive and updated synthesis of randomized evidence comparing GA and non-GA in patients undergoing EVT for AIS. By including 11 RCTs encompassing 1,674 participants, this study confirms that GA is associated with a higher rate of successful angiographic reperfusion but does not seem to improve 3-month functional independence or mortality compared with non-GA. These findings reconcile some of the inconsistencies in prior meta-analyses and clarify that while GA confers procedural advantages, these do not necessarily translate into superior patient-centered outcomes in the early recovery phase.

The observed improvement in reperfusion success under GA is consistent with several earlier analyses (22, 24), both of which found that GA facilitates more complete vessel recanalization. The most plausible explanation is that GA provides a motionless operative field, allowing for optimal fluoroscopic visualization, accurate device deployment, and avoidance of sudden patient movement during critical catheter manipulations (31, 32). Moreover, GA enables tight control of respiratory and cardiovascular parameters, particularly arterial carbon dioxide and blood pressure, which can influence cerebral perfusion pressure and the success of thrombectomy (33, 34). In contrast, conscious sedation or local anesthesia may be complicated by patient agitation or pain, occasionally requiring conversion to GA mid-procedure—an event that prolongs treatment time and may increase procedural complexity (35). The current findings thus reaffirm that procedural control under GA contributes to higher technical success in achieving reperfusion (mTICI 2b–3), although the clinical benefit remains uncertain.

Although our pooled analysis showed that GA was associated with higher rates of successful reperfusion, this finding did not consistently translate into improved functional recovery. This relationship should be interpreted cautiously, as several early RCTs demonstrated imbalances in baseline characteristics that may have biased functional outcomes against GA. Furthermore, achieving macrovascular reperfusion does not necessarily guarantee tissue-level benefit, and emerging concepts such as microvascular no-reflow, reperfusion injury, collateral failure, and persistent perfusion mismatch likely contribute to the phenomenon of “futile recanalization” (36). These mechanisms may limit the clinical impact of reperfusion irrespective of anesthesia modality and help explain the discrepancy between procedural success and patient-centered outcomes observed in our analysis. Several mechanisms may underlie this discrepancy. First, transient intraoperative hypotension, which is more common under GA, may offset the potential benefits of early reperfusion by reducing collateral circulation and worsening ischemic penumbra injury (37). Second, GA eliminates intra-procedural neurological monitoring, potentially delaying recognition of new ischemic symptoms or procedural complications (38). Third, patients randomized to GA often had more severe baseline deficits or larger ischemic cores in earlier studies, introducing a subtle residual confounding effect even within randomized designs (10). Finally, downstream factors such as delayed extubation, increased pulmonary complications, and longer intensive care stays under GA may influence early recovery trajectories (39). Thus, while GA offers technical advantages, its systemic effects may counterbalance those benefits, resulting in comparable 90-day functional outcomes between anesthetic strategies.

Comparison with previous meta-analyses further emphasizes the incremental value of this updated work. Earlier meta-analyses (20, 40) suggested that GA might improve both reperfusion and functional outcomes, but these conclusions were largely based on a smaller pool of single-center RCTs with limited sample size and significant crossover rates or observational studies, which may confound the findings. Subsequent analyses questioned the durability of these benefits, particularly when newer multicenter trials were incorporated (22, 24). However, several of these meta-analyses remained incomplete because they did not include trials published after 2023, some of which expanded the evidence base to posterior-circulation strokes and standardized hemodynamic management protocols (13, 18, 19, 25). This meta-analysis provides the most up-to-date synthesis of randomized evidence, incorporating several recent RCTs completed after prior 2023–2024 analyses. By pooling the full body of available data, our results refine the precision of effect estimates and confirm that improved reperfusion with GA does not consistently translate into better functional recovery. Importantly, the updated evidence demonstrates that the magnitude of the GA–non-GA difference is smaller than suggested in early trials, likely reflecting more standardized procedural workflows, improved hemodynamic management, and reduced confounding in modern practice. These findings offer clinicians an integrated and contemporary perspective on anesthesia choice during EVT, grounded in the totality of randomized data available to date.

The subgroup analyses offer additional insights into the generalizability of the results. The absence of significant differences between anterior and posterior circulation strokes suggests that the impact of anesthesia type on procedural and clinical outcomes is not strongly modulated by the vascular territory involved. This finding supports the notion that systemic physiological factors, rather than lesion location, primarily mediate anesthesia-related differences in EVT outcomes. Similarly, the lack of interaction with baseline NIHSS implies that the benefits and risks of GA apply across a wide range of stroke severities, reinforcing the appropriateness of individualized anesthesia selection based on patient condition and procedural context. Sensitivity analyses, which showed consistent results after sequential exclusion of individual studies, highlight the robustness of the pooled estimates and indicate that no single trial disproportionately influenced the overall conclusions. Although one trial (13) reported a relatively high crossover rate (~30%), leave-one-out analyses showed that exclusion of this study did not meaningfully alter the pooled effects, suggesting limited influence on the overall conclusions. Similarly, although CS and MAC differ in depth of sedation, MAC was used in only one RCT with substantial overlap in sedative approach (17), and sensitivity analyses excluding this study yielded consistent findings. Together, these results support the robustness of the pooled estimates comparing GA with non-GA across contemporary RCTs.

Several strengths distinguish the present meta-analysis from prior work. The study followed PRISMA and Cochrane guidelines and included only RCTs to minimize confounding. The inclusion of recently published trials ensures that the evidence reflects contemporary EVT techniques, anesthesia practices, and workflow optimizations. Rigorous quality assessment using the Cochrane RoB 2 tool showed that all included trials were of moderate methodological quality, with low risk of bias in outcome assessment and minimal attrition. Moreover, by separately analyzing angiographic, functional, and mortality outcomes, this study provides a nuanced understanding of how anesthesia influences procedural success and clinical recovery. Finally, the application of GRADE methodology to evaluate the certainty of evidence offers transparency in interpreting confidence levels for each endpoint. Nonetheless, several limitations should be acknowledged. First, despite the inclusion of recent large-scale RCTs, the total sample size remains below the optimal information threshold for definitive conclusions regarding functional outcomes, leading to downgrading for imprecision. Second, all trials were open-label by necessity, and crossover from non-GA to GA, though infrequent, could dilute between-group differences. Third, heterogeneity in anesthetic protocols, hemodynamic targets, and periprocedural management across trials may have influenced outcomes. Although all studies clearly distinguished GA from non-GA, detailed information on maintenance agents, depth of anesthesia, ventilation targets (such as ETCO₂), or hemodynamic management strategies was inconsistently reported. These factors may affect cerebral blood flow and reperfusion physiology, yet could not be systematically analyzed using study-level data. Our inclusion strategy prioritized capturing all RCTs comparing anesthesia modality, but the inability to account for protocol-level variability limits interpretation of potential mechanistic differences between GA and non-GA. Future studies with standardized or fully reported anesthetic protocols are needed to clarify these effects. A further limitation relates to crossover from CS to GA, which occurred in several trials. Although expected in clinical practice, such conversions may attenuate differences between groups. We did not exclude studies based on a strict numerical cutoff. Instead, we excluded only those in which crossover undermined the integrity of the randomized comparison. In the included RCTs, crossover was unidirectional and remained within a range that preserved the intention-to-treat contrast between GA and non-GA, but residual dilution of true effects cannot be ruled out. Indeed, because conversion typically occurs for safety reasons—such as agitation, airway compromise, or neurological deterioration—the procedural environment for these patients ultimately reflects GA rather than the randomized CS condition. As a result, any benefits of GA on procedural stability or reperfusion may be partially transferred into the CS arm, leading to dilution of between-group differences. Although all trials appropriately used intention-to-treat analyses, which preserves the validity of the randomized comparison, this crossover likely biases results toward the null and reduces the ability to detect true differences attributable to anesthesia modality. These implications have now been explicitly acknowledged, and future trials with standardized criteria for conversion and detailed reporting may help quantify the magnitude of this bias. Moreover, most studies originated from high-volume stroke centers in Europe and Asia, limiting generalizability to other healthcare systems or low-resource settings. In addition, individual patient-level data were unavailable, precluding more granular analyses of factors such as intraoperative blood pressure (BP) variability, collateral status, or time-to-reperfusion—all of which may modify the relationship between anesthesia and outcome. Particularly, there was significant heterogeneity in intra-procedural BP reporting across the included RCTs. Although several trials incorporated protocolized hemodynamic targets to minimize hypotension or excessive hypertension, the way BP data were presented varied widely—ranging from mean SBP or MAP at isolated time points, to lowest achieved MAP, to the proportion of patients experiencing a > 20% BP drop, or the duration of time spent outside predefined SBP thresholds. Other studies reported only the presence of BP protocols without providing extractable numerical values. As a result, BP data could not be summarized in a uniform format, nor could they be incorporated into quantitative analyses or used to assess whether hemodynamic control contributed to the divergence observed between procedural reperfusion rates and functional recovery. Importantly, individual patient–level data were not available, and analyses linking BP trajectories with outcomes would require granular, time-resolved physiological data. Future RCTs should adopt standardized definitions, monitoring strategies, and reporting frameworks for BP to enable more robust assessment of its role as a potential source of confounding. Finally, potential publication bias cannot be fully excluded, as neutral or positive findings may be more likely to be published in this field, although visual inspection of funnel plots did not suggest strong asymmetry.

Clinically, these findings support a pragmatic approach to anesthesia selection during EVT. Given that GA improves technical success but not short-term functional outcomes, anesthetic choice should be individualized, balancing patient cooperation, airway safety, and operator preference. GA may be favored when patient agitation, posterior-circulation occlusion, or complex anatomy threatens procedural safety, whereas non-GA remains reasonable in cooperative anterior-circulation strokes to minimize delays. Importantly, these data underscore that optimizing periprocedural physiological control—rather than simply the anesthesia modality—may hold the key to improving clinical outcomes. Future research should focus on standardized GA protocols that minimize hypotension, explore the role of conscious sedation depth monitoring, and incorporate patient-centered endpoints such as long-term cognitive recovery and quality of life. Finally, important research gaps remain, including the need for standardized intra-procedural hemodynamic targets, consistent monitoring and reporting of sedation depth, and longer-term neurological and cognitive follow-up to better understand how anesthetic strategies influence recovery trajectories, which should be also explored in future studies.

## Conclusion

In conclusion, this updated meta-analysis of RCTs demonstrates that GA significantly enhances angiographic reperfusion success but does not improve 3-month functional independence or survival compared with non-GA in patients undergoing EVT for AIS. The findings highlight the complex interplay between procedural control and systemic physiology in stroke care and emphasize the need for individualized, evidence-based anesthesia strategies. As EVT continues to evolve, integrating anesthetic management into comprehensive stroke systems of care will be crucial to translating procedural success into durable neurological recovery. On the other hand, given baseline imbalances in early trials, variability in hemodynamic management, and potential tissue-level mechanisms limiting clinical benefit, the choice of anesthesia should remain individualized until further evidence becomes available.

## Data Availability

The original contributions presented in the study are included in the article/Supplementary material, further inquiries can be directed to the corresponding author.
