# Supplementary material for: Effects of general anesthesia on short-term outcomes of patients with acute ischemic stroke after endovascular treatments: a meta-analysis
Source: Front Neurol. 2026 Jan 12;16:1728140. doi: 10.3389/fneur.2025.1728140 (PMC12852987; doi:10.3389/fneur.2025.1728140)
Supplement: Supplementary file 1 [file Table_1.DOCX]

**Supplemental File 1** Detailed search strategy for each database

**PubMed**

#1 “Anesthesia, General”[Mesh] OR “Anesthesia, Local”[Mesh] OR “Conscious Sedation”[Mesh] OR “Monitored Anesthesia Care”[Mesh]

#2 (general anesthesia[Title/Abstract] OR conscious sedation[Title/Abstract] OR local anesthesia[Title/Abstract] OR monitored anesthesia care[Title/Abstract] OR procedural sedation[Title/Abstract])

#3 “Stroke”[Mesh] OR “Brain Infarction”[Mesh] OR “Ischemic Stroke”[Mesh]

#4 (ischemic stroke[Title/Abstract] OR stroke[Title/Abstract] OR cerebral infarction[Title/Abstract] OR brain infarction[Title/Abstract] OR cerebrovascular infarction[Title/Abstract])

#5 “Endovascular Procedures”[Mesh] OR “Thrombectomy”[Mesh] OR “Catheterization, Peripheral”[Mesh]

#6 (endovascular therapy[Title/Abstract] OR mechanical thrombectomy[Title/Abstract] OR thrombectomy[Title/Abstract] OR intra-arterial thrombolysis[Title/Abstract] OR endovascular thrombectomy[Title/Abstract] OR neurointervention[Title/Abstract] OR endovascular treatment[Title/Abstract] OR contact aspiration[Title/Abstract] OR endovascular[Title/Abstract] OR stent[Title/Abstract] OR intra-arterial[Title/Abstract] OR intraarterial[Title/Abstract])

#7 (random[Title/Abstract] OR randomized[Title/Abstract] OR randomly[Title/Abstract] OR randomised[Title/Abstract] OR allocated[Title/Abstract] OR control[Title/Abstract] OR allocation[Title/Abstract])

#8: #1 OR #2

#9: #3 OR #4

#10: #5 OR #6

#11: #8 AND #9 AND #10 AND #7

**Embase**

1. ‘general anesthesia’/exp OR ‘local anesthesia’/exp OR ‘conscious sedation’/exp OR ‘monitored anesthesia care’/exp

2. general anesthesia:ab,ti OR conscious sedation:ab,ti OR local anesthesia:ab,ti OR monitored anesthesia care:ab,ti OR procedural sedation:ab,ti

3. ‘ischemic stroke’/exp OR ‘stroke’/exp OR ‘cerebral infarction’/exp OR ‘brain infarction’/exp

4. ischemic stroke:ab,ti OR stroke:ab,ti OR cerebral infarction:ab,ti OR brain infarction:ab,ti OR cerebrovascular infarction:ab,ti

5. ‘endovascular procedure’/exp OR ‘thrombectomy’/exp OR ‘intra-arterial thrombolysis’/exp OR ‘neurointervention’/exp

6. (endovascular therapy:ab,ti OR mechanical thrombectomy:ab,ti OR thrombectomy:ab,ti OR intra-arterial thrombolysis:ab,ti OR endovascular thrombectomy:ab,ti OR neurointervention:ab,ti OR endovascular treatment:ab,ti OR contact aspiration:ab,ti OR endovascular:ab,ti OR stent:ab,ti OR intra-arterial:ab,ti OR intraarterial:ab,ti)

7. random:ab,ti OR randomized:ab,ti OR randomly:ab,ti OR randomised:ab,ti OR allocated:ab,ti OR control:ab,ti OR allocation:ab,ti

8. 1 OR 2

9. 3 OR 4

10. 5 OR 6

11. 8 AND 9 AND 10 AND 7

12. limit 11 to (human and (clinical trial or randomized controlled trial))

**Web of Science**

TS = (“general anesthesia” OR “conscious sedation” OR “local anesthesia” OR “monitored anesthesia care” OR “procedural sedation”) AND TS = (“ischemic stroke” OR “stroke” OR “cerebral infarction” OR “brain infarction” OR “cerebrovascular infarction”) AND TS = (“endovascular therapy” OR “mechanical thrombectomy” OR “thrombectomy” OR “intra-arterial thrombolysis” OR “endovascular thrombectomy” OR “neurointervention” OR “endovascular treatment” OR “contact aspiration” OR “endovascular” OR “stent” OR “intra-arterial” OR “intraarterial”) AND TS = (random OR randomized OR randomly OR randomised OR allocated OR control OR allocation)

Refine by Document Type = Article, Clinical Trial, Randomized Controlled Trial

**Cochrane Library**

("general anesthesia" OR "conscious sedation" OR "local anesthesia" OR "monitored anesthesia care" OR procedural sedation) AND ("ischemic stroke" OR "stroke" OR "cerebral infarction" OR "brain infarction" OR "cerebrovascular infarction") AND ("endovascular therapy" OR "mechanical thrombectomy" OR "thrombectomy" OR "intra-arterial thrombolysis" OR "endovascular thrombectomy" OR "neurointervention" OR "endovascular treatment" OR "contact aspiration" OR "endovascular" OR "stent" OR "intra-arterial" OR "intraarterial") AND (random OR randomized OR randomly OR randomised OR allocated OR control OR allocation)
